# On the Therapeutic Forces

**Published:** 1879-01

**Authors:** 


*On the Therapeutic Forces:* An effort to consider the action of medicines in the light of the modern doctrine of the conservation of force. By Thomas J. Mays, M. D., Member of the Luzerne County Medical Society; Member of the Pennsylvania Medical Society, etc. Philadelphia: Lindsay & Blakiston. 1878.

This is an ingeniously written speculative essay upon Medicine as a *force*. The scope and intent of the work being very fully expressed by the following "Conclusion:"

In the foregoing chapters I have considered examples of the three great general classes of therapeutic agents, viz., the chemical and mechanical stimulants, and narcotics, and think that I have at least given some reason for the belief that all these varied, and in some cases opposite phenomena which are produced by these agents, are but the result of the action of forces, and, before coming to a close, I will make a brief recapitulation, and point out certain conclusions to which these deductions lead.

The animal body, then, is composed of organic molecules which in health are in a constant state of activity. This molecular activity is maintained in the natural way by chemical stimulants, substances which undergo oxidation in the body, and thus give free their force. In diseases, however, the ordinary chemical stimulants are not always capable of furnishing the requisite degree of molecular activity to the body; hence they are reinforced by others, like alcohol, the hypophosphites, etc., which possess a superior capacity for undergoing combustion. On the other hand the molecular activity of the body may also be enhanced by the mechanical stimulants—substances like quinia, ammonia, etc., which transmit their motion directly to the body without undergoing previous oxidation, and these are powerful adjuvants to the chemical stimulants in treating disease. And, again, we have especial mechanical stimulants of the nervous system, like opium in small doses, as well as special mechanical depressants of this tract of bodily tissue, like opium and alcohol in large doses. We thus find, then, that these three general classes comprise the action of many of our most reliable and important remedies, and it might easily be shown that the therapeutic action of many other substances would become intelligible if considered in a similar light.

By regarding our remedial agents as the embodiment of forces we can readily account for the cause of the great difference in the effects of small and large doses of medicines. It must, however, not be imagined that this twofold action of medicines is confined to those which have been discussed in this paper, for it will be found that the majority, if not all of our therapeutic measures are subject to the same law. It will, also, from what has been said, be seen how unfair and even how censurable it is to define the action of a medicine without at the same time qualifying the quantity and the bodily conditions under which it so acts. It would be just as unwise for one to make the broad assertion that ipecacuanha is an emetic, while every one knows that in small doses it also relieves emesis and it is equally well known that in doses in which it ordinarily produces vomiting, it is also very efficient in curing dysentery without displaying much, if any, of its emetic properties; and moreover, the recent researches of Dr. Rutherford and M. Vignal shows it to be a most powerful cholagogue, superior even to calomel and podophyllin. This is likewise true of aloes, for in large doses this substance possesses a decided cathartic action on the lower bowel, but in small doses it is also a tonic to this section of the alimentary canal. Instances of a similar character might be multiplied, but they would only serve to still further demonstrate the careless and unscientific spirit with which we treat our therapeutic forces.

From the foregoing, we likewise learn that all therapeutic forces when viewed from the standpoint of life, must either move in harmony with or in opposition to the vital forces. But we have also seen on the one hand that there are medicinal forces, of which quinia affords an example, which move in opposition to life generally, and do yet in small quantities have the power of enhancing the life process; while, on the other hand, there are others, like alcohol, which move harmoniously with the bodily forces in small quantities, but in large doses they become powerfully antagonistic to the latter. Hence, the chief and only true aim in the investigation of the action of therapeutic forces, consists, not merely in determining the action of each particular medicine, but the *degree* of force in each medicine which promotes or retards the bodlly forces, *i. e*,, the action of small and large doses. But since therapeutics is, or ought to be, the science and art of curing disease, it follows that a complete knowledge of the action of these forces cannot be gained by a study of their effects on the healthy body, but these must be confirmed by observation and experience at the bedside, although much can be learned in a general way from physiological experiments or "provings" on the healthy human system, as well as on that of the lower animals. Indeed, one of the most promising avenues to the determination of the action of medicines consists in studying the influence of these agents on disease induced artificially in the lower animals.

I think these deductions illustrate the fact that the equality of cause and effect is as true when applied to therapeutics as to any other department of nature, and they furthermore impress on us more than ever the stern truth, that the medical man in pursuing his calling has to deal with the most intricate and delicate forces, which are potent for good or evil, and which require to be regulated by the most consummate skill and enlightened judgment. And, finally, I indulge in the hope that this feeble effort in placing the study of therapeutics on a firmer basis than it has heretofore occupied, may give an impulse to future inquiry in this direction ; for just as soon as the medical profession rightly appreciates that the art of curing disease is controlled by invariable law, so soon will it be wrenched from the uncertainty of empiricism* and of charlatanism, and rise to the dignity of a science.

* It is far from my intention to underrate the valuable influence of medical empiricism, for to do so would be but heaping undeserved reproach on "the bridge that carried us over." One cannot call it an inheritance of "unalloyed blessedness," yet it certainly had its good side also.

